# Pan-Cancer Analysis of Mitochondria Chaperone-Client Co-Expression Reveals Chaperone Functional Partitioning

**DOI:** 10.3390/cancers12040825

**Published:** 2020-03-30

**Authors:** Geut Galai, Hila Ben-David, Liron Levin, Martin F. Orth, Thomas G. P. Grünewald, Shai Pilosof, Shimon Bershtein, Barak Rotblat

**Affiliations:** 1 Department of Life Sciences, Ben-Gurion University of the Negev, Beer Sheva 8410501, Israel; geutg@post.bgu.ac.il (G.G.); shainova@gmail.com (S.P.); 2 The National Institute for Biotechnology in the Negev, Beer Sheva 8410501, Israel; levinl@post.bgu.ac.il; 3 Max-Eder Research Group for Pediatric Sarcoma Biology, Institute of Pathology of the LMU Munich, 80337 Munich, Germany; martin.orth@med.uni-muenchen.de (M.F.O.); t.gruenewald@dkfz-heidelberg.de (T.G.P.G.); 4 Institute of Pathology of the LMU Munich, 80337 Munich, Germany; 5 Division of Translational Pediatric Sarcoma Research, German Cancer Research Center (DKFZ), 69120 Heidelberg, Germany; 6 Hopp Children’s Cancer Center Heidelberg (KiTZ), 69120 Heidelberg, Germany; 7 German Cancer Consortium (DKTK), 69120 Heidelberg, Germany

**Keywords:** cancer, co-expression, chaperone, mitochondria, bioinformatics analysis

## Abstract

Metabolic reprogramming is a hallmark of cancer. Such reprogramming entails the up-regulation of the expression of specific mitochondrial proteins, thus increasing the burden on the mitochondrial protein quality control. However, very little is known about the specificity of interactions between mitochondrial chaperones and their clients, or to what extent the mitochondrial chaperone–client co-expression is coordinated. We hypothesized that a physical interaction between a chaperone and its client in mitochondria ought to be manifested in the co-expression pattern of both transcripts. Using The Cancer Genome Atlas (TCGA) gene expression data from 13 tumor entities, we constructed the mitochondrial chaperone-client co-expression network. We determined that the network is comprised of three distinct modules, each populated with unique chaperone-clients co-expression pairs belonging to distinct functional groups. Surprisingly, chaperonins *HSPD1* and *HSPE1*, which are known to comprise a functional complex, each occupied a different module: *HSPD1* co-expressed with tricarboxylic acid cycle cycle enzymes, while *HSPE1* co-expressed with proteins involved in oxidative phosphorylation. Importantly, we found that the genes in each module were enriched for discrete transcription factor binding sites, suggesting the mechanism for the coordinated co-expression. We propose that our mitochondrial chaperone–client interactome can facilitate the identification of chaperones supporting specific mitochondrial pathways and bring forth a fundamental principle in metabolic adaptation.

## 1. Introduction

Metabolic reprogramming is a hallmark of cancer [1]. It plays a fundamental role in many—if not all—aspects of the disease, including cell transformation [2,3,4], oncogenic signaling [5,6,7], adaptation to the tumor micro-environment [8,9], drug resistance [10,11,12,13,14], and metastasis [15,16,17,18,19,20,21,22,23,24]. Interestingly, mutations in metabolic genes such as *IDH1* [25,26] are rare, and in the majority of cases, metabolic reprogramming occurs at the level of gene expression, leading to changes in the effective concentration of specific metabolic enzymes [27,28,29,30,31,32]. 

Due to the glycolytic nature of most tumors, it was thought that mitochondria are dispensable for tumor cells [31]. However, our current understanding completely upends this traditional view [33]. Indeed, multiple studies have demonstrated that tumor cells are dependent on mitochondria [34,35,36]. This dependency is particularly crucial for tumor cells experiencing glucose starvation [37,38]. 

The mitochondria are remnants of the “fusion event” and harbor bacterial-like genes in their genome [39]. While 13 mitochondrial proteins are known to be synthetized in the mitochondria [40], the majority of over 1000 mitochondrial proteins are encoded in the nucleus, synthetized by cytosolic ribosomes, and imported into the mitochondria [41,42]. These import processes, as well as the mitochondrial redox state and elevated intra-mitochondrial temperature [43], present a formidable stress on protein homeostasis within mitochondria. To meet this challenge, mitochondria are equipped with molecular chaperones (chaperones and proteases) that ensure protein quality control by assisting, on one hand, in translocating and folding of the imported proteins, and, on the other hand, in the degradation and removal of aggregated and damaged proteins [44].

Recent studies revealed that chaperones fold specific proteins with different efficiencies, and that cells fine-tune the expression of chaperones to meet the demand for client folding. For example, in *C. elegans*, *Unc-45* is a myosin-specific chaperone [45], and it is co-expressed with myosin during muscle development [46]. In mammalian cells, the topology of the mitochondrial chaperone–client network is not fully known, nor it is known whether chaperone–client expression is coordinated in tumor cells to support metabolic adaptation.

Here, we use tumor gene expression (RNA-seq) data from The Cancer Genome Atlas (TCGA) to identify the mitochondrial chaperone–client network. We found that specific chaperone–client co-expression pairs form three distinct modules within the network. Genes in each module belong to distinct functional pathways and are associated with a common set of transcription factor binding sites in their promoters. These findings support a model in which metabolic reprogramming in cancer requires coordination regarding the expression of mitochondrial proteins and their specific chaperones.

## 2. Results

### 2.1. Mitochondrial Chaperone–Client Co-Expression Patterns in Cancer

**Figure 1:**
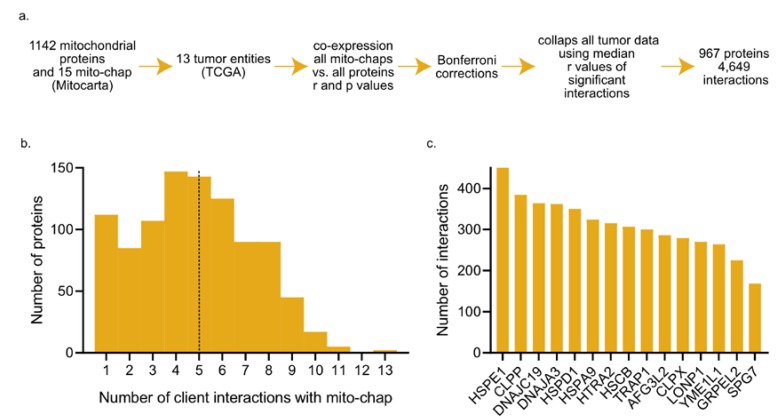
(**a**) Workflow scheme. (**b**) Distribution of the number of interactions with chaperones, dashed line depicts the median number of interactions per protein. (**c**) The number of interactions of each mitochondrial chaperone (mito-chap).

Since the co-expression of a given mitochondrial chaperone and its client is indicative of chaperone–client interaction (CCI), we assembled a candidate list of CCIs using the mitochondrial chaperone and co-chaperone list comprised by Voos [44] (Table S1; 15 chaperones) and the mitochondrial proteins list in MitoCarta [42,47] (Table S1; 1142 proteins). Together, we analyzed 13 cancer entities (listed in Table S1) and used a Spearman correlation test to measure co-expression (r and *p* values are listed in Tables S2 and S3; the r-value distributions, including negative values, are shown in Figure S1). We only used positive correlations because negative values are not indicative of CCI. We expected that each cancer type will exhibit different co-expression patterns, and indeed, this is the case (Figure S2). Nevertheless, we hypothesize that in case the folding of a particular substrate is very much dependent on the expression of a specific chaperone, co-expression between the two will be found in multiple cancers. To gain a broad view of co-expression patterns, beyond an interaction occurring in one, or very few, particular cancer types, we combined all tumor co-expression data by using the interaction’s median r-values (Table S4). In tumor entities in which a particular interaction (co-expression between a chaperone and client) did not reach statistical significance, following Bonferroni correction, we assigned this interaction a score of zero (Table S4). As a cutoff, the original *p*-value set was 0.05, and after Bonferroni correction, it was 2.9 × 10^−6^. Collectively, 967 proteins participating in 4649 interactions reached statistical significance and were used in the following analyses (Figure 1a). We found that 50% of proteins are co-expressed with less than 5 mito-chaperones (Figure 1b), and that most mitochondrial chaperones interact with less than 300 clients (Figure 1c). In addition, we preformed pan-cancer co-expression analysis using two other *p*-value cutoffs and found that the distributions were not qualitatively affected (Figure S3), suggesting that the distributions are not *p*-value specific. We speculated that during metabolic reprograming, the cell up-regulates all mitochondrial proteins together; however, not all mitochondrial chaperones are co-expressed with all mitochondrial proteins, suggesting that there is a degree of specificity in CCIs in the mitochondria.

### 2.2. Mitochondrial Chaperone–Client Network is Composed of Three Distinct Modules

**Figure 2:**
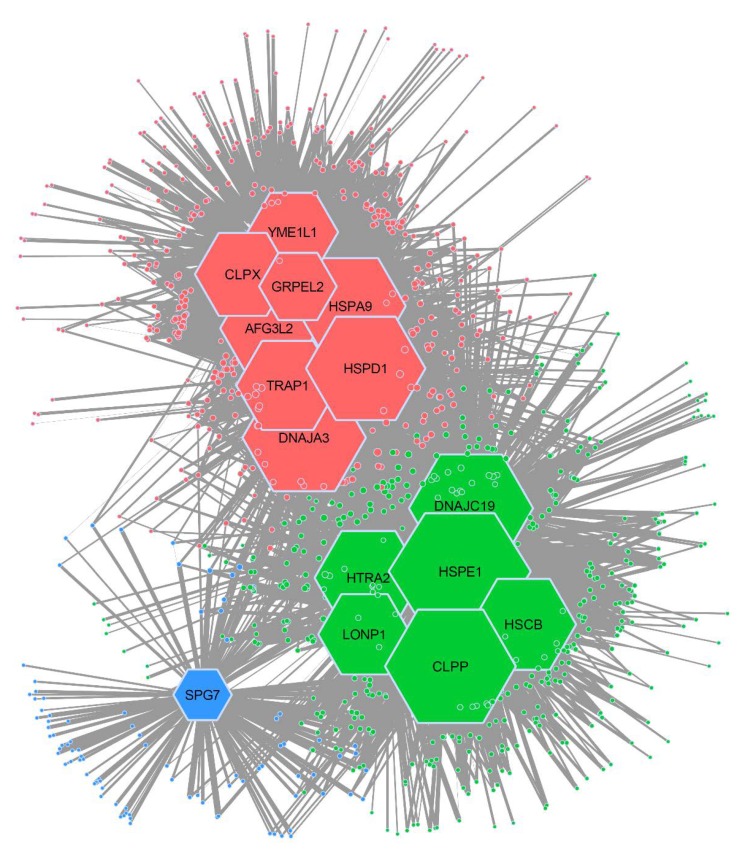
Network analysis of mitochondrial chaperone–client co-expression. An Infomap tool was used to analyze the structure of the mitochondrial chaperone–client co-expression network, revealing three distinct modules. Module HSPD1 is depicted in red; module HSPE1 is depicted in green; and module SPG7 is depicted in blue. Size of the chaperone reflects the number of interactions. Modules are named after the chaperone with most interactions.

We used our co-expression data to build the CCI network and to infer its topology. To this end, we performed community detection analysis (Figure 2) and found that our network is non-random (Figure S4). Specifically, the network has a non-random modular structure (*p*-value < 0.001) composed of three distinct modules. Each module is comprised of chaperones and clients that are co-expressed more strongly with each other than with chaperones or clients from other modules. We named these modules HSPD1, HSPE1, and SPG7, and marked them as red, green, and blue in Figure 2, respectively. Module HSPD1 is comprised of eight chaperones and 422 clients. Module HSPE1 is comprised of six chaperones and the largest number of clients (451). SPG7 is the smallest module with 94 clients and only one chaperone. The remaining 176 proteins could not be associated with any module due to a lack of co-expression with any of the chaperones.

**Figure 3:**
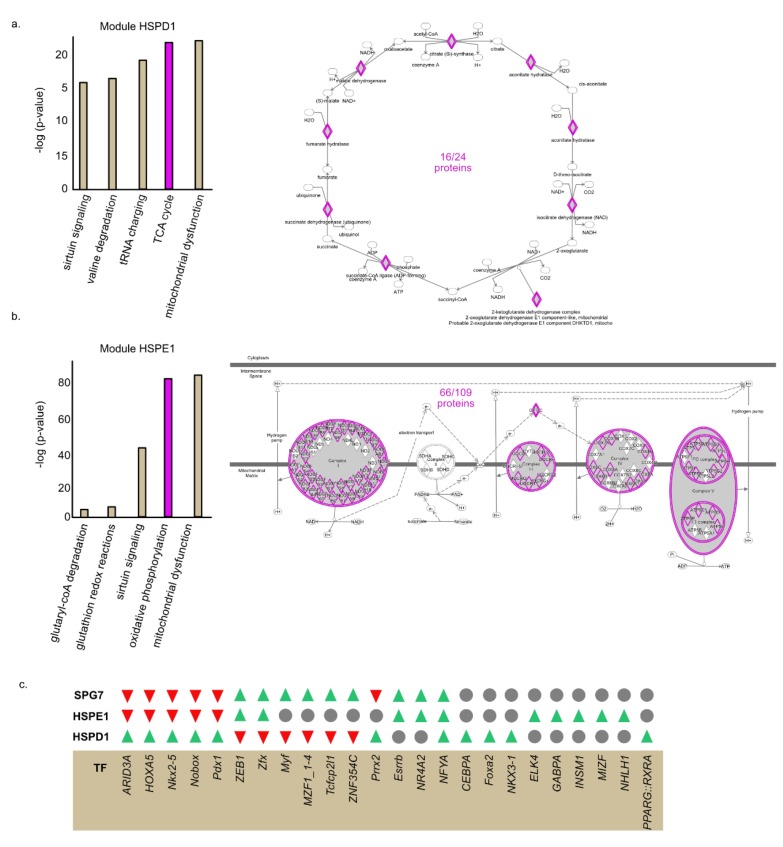
Proteins in each module are enriched for distinct mitochondrial pathways and distinct transcription factor motifs in their promoters. a, b. proteins belonging to each module were interrogated using Ingenuity Pathway Analysis (IPA) to identify biological pathways enriched in each list of proteins. (**a**) Module HSPD1 (**b**) Module HSPE1. (**c**) Transcription factor binding sites enriched or depleted in each module were identified using oPOSSUM analysis. Green = enriched in the module; Red = depleted in the module; Gray = not enriched or depleted in the module.

Next, we used Ingenuity Pathway Analysis (IPA) to determine whether genes co-expressed within each module are enriched with distinct cellular pathways. We found that chaperones within module HSPD1 are co-expressed with genes belonging to TCA cycle genes (Figure 3a). In contrast, the chaperones of module HSPE1 are co-expressed with genes belonging to the OXPHOS complexes (Figure 3b). Module SPG7 transcripts are associated with different mitochondrial pathways with low number of proteins in each category (Table S5). These findings support a model in which proteins functioning in distinct mitochondrial pathways are dependent on a particular set of chaperones for their folding and that their expression is coordinated. 

### 2.3. Common TF Binding Sites in the Promoters of Clustered Proteins

We found distinct sets of chaperone and clients who are co-expressed with one another. Common transcription regulation can provide a possible explanation for co-expression. We asked whether genes found in the same module share similar transcription factor (TF) binding sites in their promoters. To address this question, we used oPOSSUM analysis [48,49,50] and identified TF binding sites enriched or depleted in the list of gene of each module (Table S6). We focused on the top and bottom 10 TF (according to their z-scores) (Figure 3c). Interestingly, we found that module HSPD1 and module HSPE1 genes are enriched for distinct TF binding sites (Figure 3c). Remarkably, we found that some TF binding sites that are enriched in one module are significantly depleted from the other and vice versa. For example, the AT-Rich Interaction Domain 3A (ARID3A) binding site is enriched in genes comprising module HSPD1 but depleted in genes belonging to modules HSPE1 and SPG7. Furthermore, the Zinc Finger Protein X-Linked (Zfx) binding site is enriched in the promoters of modules HSPE1 and SPG7 genes but depleted from module HSPD1 genes. In sum, these results suggest that the network topology is encoded in the promoters of mitochondrial genes.

### 2.4. Chaperone–Chaperone Co-Expression Recapitulates Chaperone–Client Co-Expression

**Figure 4:**
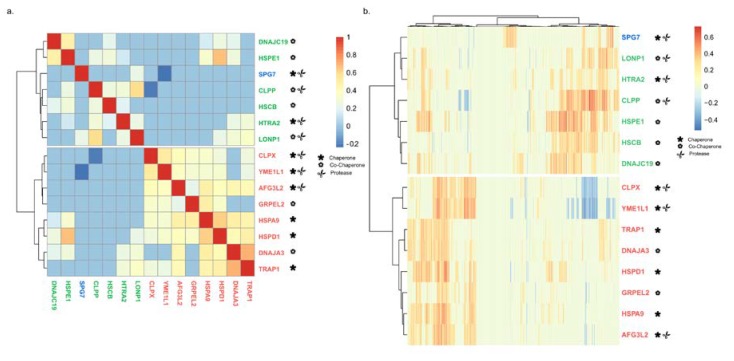
Major clustering groups similar to the generated modules. (**a**) Hierarchical clustering of chaperone–chaperone interactions. (**b**) Hierarchical clustering of chaperone–client interactions. r-values are color-coded. Chaperones are color-coded according to their module.

We next looked into the co-expression patterns of mitochondrial chaperones with each other by using hierarchical clustering. This analysis clusters mitochondrial chaperones according to their interactions with other mitochondrial chaperones in such a way that the chaperones with similar co-expression patterns are found closer together (Figure 4a). As anticipated from the functional interaction between chaperones and co-chaperones, *HSPA9* (Hsp70) was clustered with its cognate co-chaperones *DNAJA3* and *GRPEL3*. However, in the case of other known chaperone–co-chaperone functional complexes, such as *HSPD1* and *HSPE1*, and *CLPX* and *CLPP*, hierarchical clustering placed chaperones and co-chaperones in different functional groups, despite the fact that they are strongly co-expressed with each other (Figure 4a). These findings hint at the possibility that chaperones and co-chaperones function in distinct biological processes.

We noted that the two major groups in our hierarchical clustering (Figure 4a) are similar in composition to the network modules HSPD1 and HSPE1 that were obtained through the co-expression analysis of chaperones with 1142 transcripts (Figure 2). Yet, *SPG7* was clustered differently from its position in the network module. We wondered to what extent the chaperone–chaperone co-expression hierarchical clustering is maintained in chaperone–client co-expression hierarchical clustering (Figure 4b). In accord with our chaperone–chaperone co-expression analysis (Figure 4a), we found that the chaperones are separated into two similar major groups (Figure 4b). Therefore, we conclude that the information found in the chaperone–chaperone co-expression network is sufficient to generate the two major groups obtained using chaperone–client co-expression analysis. 

### 2.5. HSPE1 and HSPD1 Exhibit Differential Co-Expression Patterns 

The chaperones with the highest number of interactions in their module are *HSPD1* and *HSPE1*. *HSPD1* and *HSPE1* are the mammalian homologues of the bacterial chaperonin *GroEL/ES* complex [43]. GroEL forms back-to-back stacked double-ring structures that provide chambers allowing client proteins to fold in isolation. The chambers close through binding the co-chaperone GroES [46].

**Figure 5:**
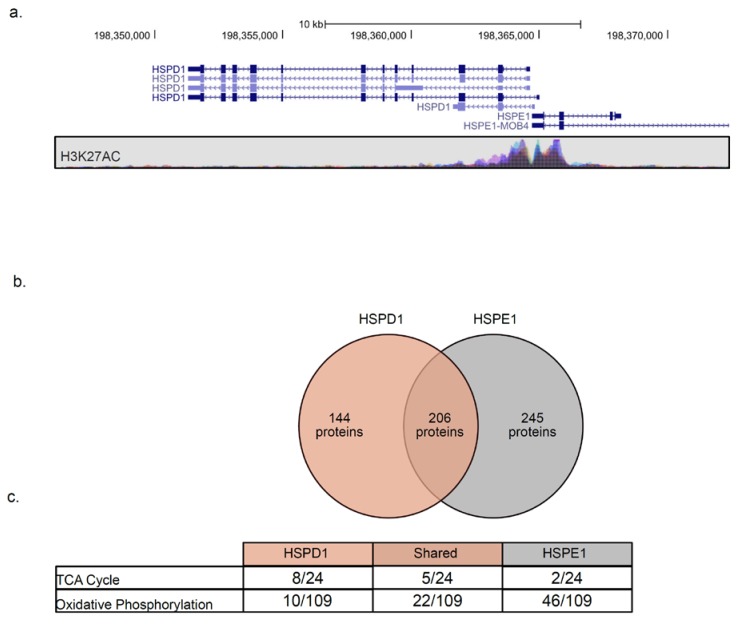
*HSPD1* and *HSPE1* share a promoter but are co-expressed with distinct mitochondrial proteins. (**a**) The genomic location and K27acetylation of *HSPD1* and *HSPE1* were obtained from the University of California Santa Cruze (UCSC) genome browser. (**b**) Venn diagram representing proteins co-expressed with each chaperone. (**c**) IPA analysis of each group of proteins identified in b depicts the number of proteins found to belong to the specific pathway and the total number of proteins in the pathway.

In bacterial genomes, genes encoding *GroEL* and *GroES* are placed under the same operon, ensuring co-expression. In the human genome, *HSPD1* and *HSPE1* are found in a head-to-head orientation and share a bi-directional promoter [47], as indicated by the histone 3 lysine 27 acetylation (H3K27ac) pattern (Figure 5a). As expected, the expression of the two genes is highly correlated (Figure 4a). Surprisingly, *HSPD1* and *HSPE1* are co-expressed with different sets of mitochondrial proteins (Figure 4b) and mito-chaperones (Figure 4a). We compared the list of genes co-expressed with each chaperonin and found that 206 proteins are co-expressed with both chaperones, 144 are co-expressed only with *HSPD1*, and 245 are co-expressed only with *HSPE1* (Figure 5b, Table S4). Using IPA, we found that proteins co-expressed with *HSPD1* or *HSPE1* belong to different mitochondrial pathways. Namely, tricarboxylic acid cycle (TCA) cycle enzymes are mostly co-expressed with *HSPD1*, while oxidative phosphorylation (OXPHOS) proteins are mostly co-expressed with *HSPE1* (Figure 5c). These findings are in line with the results obtained using network analysis, where *HSPD1* and *HSPE1* are found in distinct modules (Figure 3). Together, these findings suggest that *HSPD1* might support the folding of TCA cycle proteins, whereas *HSPE1* assists in the targeting and folding of OXPHOS proteins.

## 3. Discussion

Of over 1000 proteins found in human mitochondria [42], only 13 are encoded by the mitochondrial genome [40]. The remaining proteins are encoded by nuclear genes, synthesized by cytoplasmic ribosomes, and transported into the mitochondrial matrix via an intricate translocation machinery [41,42]. Due to the small dimensions of the translocation channels, proteins destined for mitochondria are translocated via a double membrane in a mostly unfolded state [41]. Molecular chaperones play a central role in the biogenesis of mitochondrial proteins by directly assisting in their transmembrane translocation, folding into an active state, and maintenance of the folded proteins in the adverse environment of the organelles [44].

Previous studies demonstrated that major bacterial chaperones (i.e., Hsp60 and Hsp70) cater to a large number of overlapping substrates [51]. Further, chaperones were shown to cooperate in interconnected networks, in which a single client can be transferred between multiple chaperones [44]. Given the inherent promiscuity of the CCIs and the interconnectivity of the chaperone network in bacteria, here we aimed at determining the topology of the chaperone–client network in mitochondria that share a common evolutionary ancestry with bacteria [39]. In particular, we wondered whether it is possible to unveil the patterns of specific CCIs within a network that is apparently dominated by promiscuous interactions. It was previously reported that specific interactions between HSP90 and CYPD, VDAC1 and ANT, and HSPD1 and CypD in mitochondria directly contribute to survival of tumor cells [52]. Therefore, we reasoned that the abundant co-expression data available from cancer tissues of multiple origins is most suitable to address this question. We turned to TCGA, extracted co-expression data for mitochondrial proteins, and performed pan-cancer analysis on 13 cancer entities. 

Our analysis revealed that most mitochondrial chaperones are co-expressed and potentially interact with over 200 clients. *HSPE1* exhibits the leading number of interactions (over 400) (Figure 1c). Surprisingly, despite the apparent promiscuity in CCIs, community detection analysis unveiled a triple module structure of the mitochondrial chaperone-client network, which is indicative of specific interactions (Figure 2). Each of the three modules is populated with chaperone–client co-expression pairs that co-express more strongly with each other than with chaperones or clients from other modules. Furthermore, the grouping of CCIs within each of the two major modules (HSPE1 and HSPD1) is associated with specific mitochondrial functional pathways. Most notably, genes associated with the HSPD1 module are enriched with TCA cycle proteins, whereas the genes of the HSPE1 module are dominated by proteins involved in oxidative phosphorylation (Figure 3). Furthermore, these interactions were also found by individually analyzing the CCI of *HSPD1* and *HSPE1*. Interestingly, most TCA cycle proteins are soluble, whereas most OXPHOS proteins are membrane bound (Figure 3). Therefore, it is tempting to speculate that *HSPD1* and *HSPE1* have acquired divergent functions, where *HSPD1* is more important for the folding of soluble proteins and *HSPE1* is more important for the folding of membrane-bound proteins.

What is the mechanism underlying the coordination of the specific co-expression patterns, as unveiled by our analysis? We reasoned that one of the mechanisms that can support the differential co-expression patterns might be a unique set of transcription factors “allocated” for specific chaperone–client expression pairs. To test this hypothesis, we determined the composition of transcription factor binding sites in the promoters of genes involved in specific chaperone–client interactions. Remarkably, we found a strong signal in the differences of the composition of specific transcription factors between genes belonging to distinct modules. For instance, the promoters of genes comprising module HSPD1 are enriched with binding sites for transcription factor *ARID3A*. However, the binding sites for this transcription factor are depleted in genes belonging to module HSPE1 (Figure 3c). This finding is far from being trivial, as both *HSPD1* and *HSPE1* genes share a common promoter/regulatory region and are strongly co-expressed (Figure 4a). Nonetheless, in addition to the common clients, each of the chaperones cater to a unique set of clients that are involved in distinct functional pathways. Our data strongly hint at the possibility that the coordination of the co-expression of chaperones with their specific clients is achieved via a shared set of transcription factor binding sites.

One of the intriguing findings of our work is the partitioning of chaperones and their cognate co-chaperones into distinct modules. Specifically, the Hsp60 chaperonin (HSPD1) is separated from its co-chaperone Hsp10 (HSPE1), and ClpX AAA+ ATPase is separated from its cognate ClpP protease. It has long been demonstrated that not all Hsp60 clients obligatory require Hsp10 co-chaperone for successful folding [51]. However, chaperone activity uniquely associated with Cpn10 was not demonstrated. Our analysis suggests a unique role of Hsp10 in chaperoning a specific set of mitochondrial proteins. Interestingly, many of the unique clients of *HSPE1* and *ClpX* belong to proteins of oxidative phosphorylation, pointing to a direct and specific involvement of these chaperones in the folding and maintenance of mitochondrial proteins destined for membrane integration.

In our analysis of the mitochondrial chaperone–client interactions, we focused on the topology of the network at a global scale. However, we also believe that our analysis will constitute a valuable resource for the identification of potential specific CCIs in cancer and normal cells. We validate this claim by testing the aforementioned interactions, which are known to be important in the growth of cancer cells [52]. Similarly to the reported interactions, we find that *TRAP1* interacts with *CypD* and *VDAC1* (but not *ANT*) in our pan-cancer analysis. Further, *HSPD1* interacts with *CypD*, as reported (Table S4) [53].

## 4. Materials and Methods 

### 4.1. Co-Expression Analysis

Gene-level transcriptome profiling (RNA-Seq) data (in the form of HTSeq–FPKM) from TCGA was download using the Genomic Data Commons Data Portal (https://portal.gdc.cancer.gov). In each tissue, the Spearman correlation was calculated between a set of 15 mitochondrial chaperone genes and 1142 genes belonging to their potential substrates. This analysis was performed for cancer tissue samples. Gene pairs that were not found to be significantly correlated (after correction for multiple testing using the Bonferroni method = 2.9 × 10^−6^) were considered to have a correlation coefficient of 0.

### 4.2. Pan-Cancer Analysis

The correlation data from the co-expression analysis for the 13 tissue types were used. For every chaperone–client interaction, or chaperone–chaperone interaction, we took the median value of the 13 tissue types and built a new co-expression table of mitochondrial chaperones against mitochondrial proteins.

### 4.3 Analysis of a Chaperone–Client Network

Using a matrix of co-expression data built from the pan-cancer analysis results, a bipartite network was built in which associations between chaperones and proteins (node) were encoded as the median co-expression (weighted edges). We only used positive co-expression values. This network was used as input for the community detection tool Infomap [54]. 

Infomap is a flow-based method that finds optimal network partitioning based on the movement of a random walker on the network to detect a module. For a given partition of the network, the random walker moves across nodes in a way that depends on the direction and weight of the edges, and it tends to stay longer in dense areas representing modules of highly connected species. The time spent in each module is converted to an information–theoretic currency by using an objective function called the map equation, which is denoted as L. The optimal network partition is the one that minimizes the value of L [55,56]. Since any community detection method will find some partition, we tested if the partition obtained for the observed network can also be obtained via random chaperone–client associations by running Infomap (and calculating L) on 1000 randomized versions of the observed network, as is commonly done in the analysis of ecological networks [57,58,59,60]. The randomization was done in two steps. First, we shuffled matrix cells but fixed the marginal row and column sums. This stringent method called the ‘curveball’ algorithm [61] limits the number of client associations (for chaperones) and the number of chaperone associations (for proteins) to that observed in nature. Then, we randomly re-distributed the co-expression correlation values for every cell in which an interaction existed. The statistical significance of the analysis (one-tailed test *p*-value) was calculated as the probability of obtaining a random network with a better partition than the observed one, as follows [50,51]:

(1) $$p=\frac{count\left(L_{sim\;i\;}>L_{obs}\right)}{N_{simulations}}$$

For visualization we used Cytoscape [62].

### 4.4. Ingenuity Pathway Analysis (IPA)

Lists of proteins associated with each module were uploaded to Ingenuity Pathway Analysis (QIAGEN) and submitted to core analysis of the canonical pathway to identify common pathways involving the clustered proteins.

### 4.5. Code

Correlation and pan-cancer analyses were performed using Python programming language (version 2.7.15 [62]) within a windows environment, with aid of the ‘pandas’ and ‘numpy’ packages. Infomap analysis and validation were performed using R (version 3.6.1 [63]) within the Linux environment with aid of the ‘vegan’ package (2.6 [64]) for the network simulation. We provide the R code and data in https://github.com/RotblatLab/MitoChapCorrelations.

### 4.6. oPOSSUM Analysis

We used the default settings in the Single Site Analysis tab (in http://opossum.cisreg.ca/oPOSSUM3/) to of analyze the list of proteins in the different modules. The results were sorted according to the z-score; the top-10 TF binding sites and the bottom 10 binding sites (all receiving negative z-scores) were used. 

## 5. Conclusions

Our analysis reveals an unexpected chaperone–client co-expression network topology where distinct set of chaperones are co-expressed with a distinct set of proteins, each belonging to a particular mitochondrial pathway. Furthermore, we found that, in some cases, chaperones and co-chaperones are found in distinct network modules, suggesting partitioning in function.

## Supplementary Materials

The following are available online at https://www.mdpi.com/2072-6694/12/4/825/s1, Figure S1: R values histogram, Figure S2: Per cancer CCI heat maps, Figure S3: Pan-cancer co-expression analysis using two other *p*-value, Figure S4: Network randomization analysis. Table S1: Genes analyzed, Table S2: Raw R values, Table S3: Raw *p* values, Table S4: Pan-cancer analysis, Table S5: Clustering and IPA data, Table S6: oPOSSUM data.
