# Supplementary material for: Pan-Cancer Analysis of Mitochondria Chaperone-Client Co-Expression Reveals Chaperone Functional Partitioning
Source: Cancers (Basel). 2020 Mar 30;12(4):825. doi: 10.3390/cancers12040825 (PMC7226338; doi:10.3390/cancers12040825)
Supplement: Supplementary file 1 [file cancers-12-00825-s001.zip › cancers-735738-supplementary/Galai_et_al_mito-chap - Revised Supplementary Files/Supplementary Figures/Supplementary Figure legend.docx]

**Supplementary Figure legend**

**Supplementary Figure 1.** The distribution of the significant pan-cancer r values. A histogram composed of the median R values calculated on the pan-cancer analysis. Only significant chaperon-client interactions are included.

**Supplementary Figure 2.** Single tissue entity R values heatmap for every cancer tissue used. Included are heatmaps for 13 cancer entities. Heatmaps show r values and are color-coded. Hierarchal clustering was done by chaperones.

**Supplementary Figure 3.** Pan cancer co-expression analysis is not p-value specific. Pan-cancer co-expression analysis was performed using additional p-value cutoffs. (a) P-value =6 x 10-7. Left: Distribution of the number of interactions with chaperones dashed line depicts the median number of interactions per protein. Right: The number of interactions of each mitochondrial chaperone (mito-chap). (b) the same as in (a) using p value=5.8 x 10-6

**Supplementary Figure 4.** Comparing the clustering analysis to randomized simulations shows a statistically significant difference. Curveball algorithm was used to create 1000 randomized networks based on the pan-cancer co-expression data. The histogram depicts L values generated by Infomap simulations of the 1000 networks. Comparing the simulated L values to the pan-cancer clustering L value shows a statistically significant difference between the two with a p-value < 0.001.
