# Supplementary material for: Pan-Cancer Analysis of Mitochondria Chaperone-Client Co-Expression Reveals Chaperone Functional Partitioning
Source: Cancers (Basel). 2020 Mar 30;12(4):825. doi: 10.3390/cancers12040825 (PMC7226338; doi:10.3390/cancers12040825)
Supplement: Supplementary file 1 [file cancers-12-00825-s001.zip › cancers-735738-supplementary/Galai_et_al_mito-chap - Revised Supplementary Files/Supplementary Figures/SUP_Fig_2_all heatmaps together.pdf]

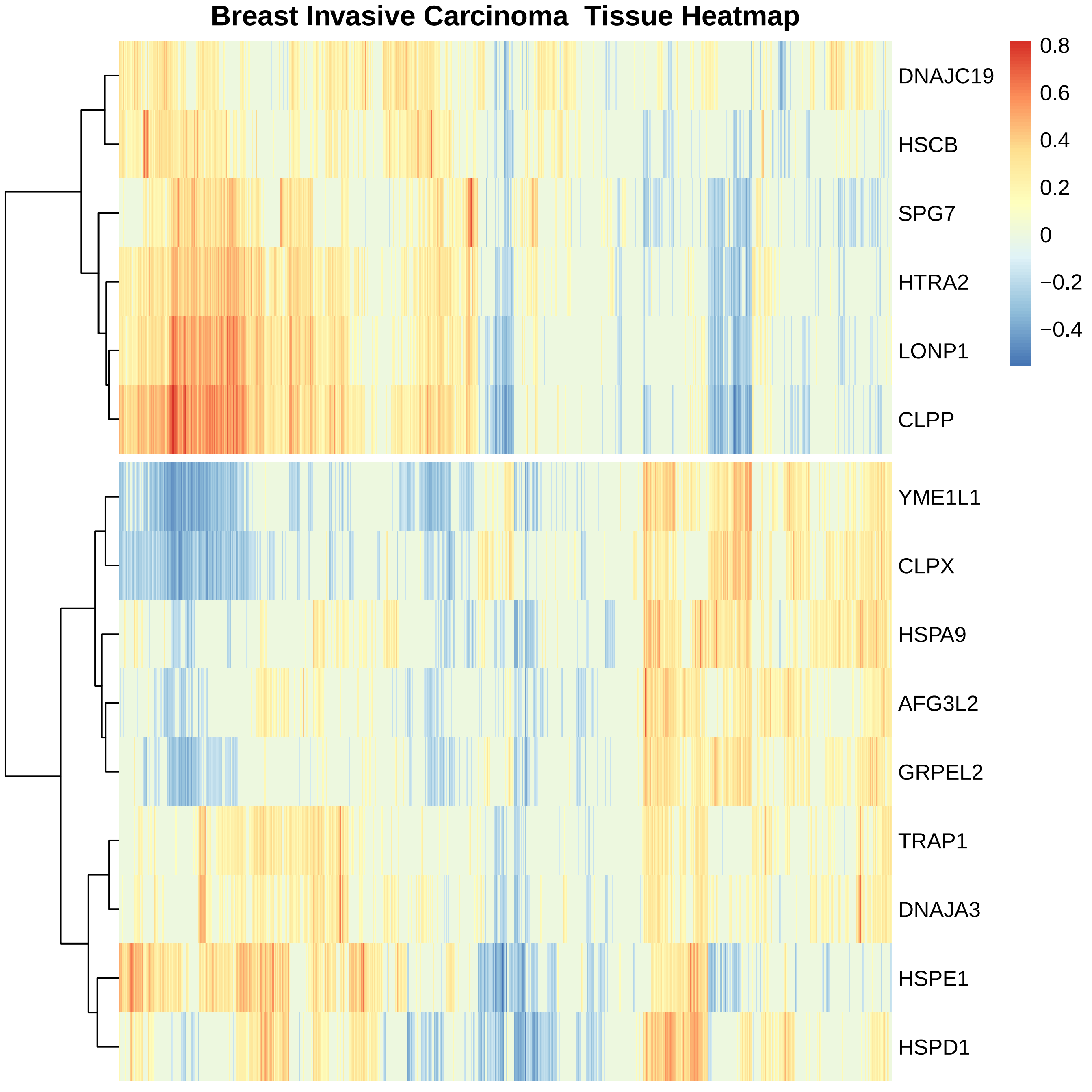

Breast Invasive Carcinoma Tissue Heatmap
DNAJC19
HSCB
SPG7
HTRA2
LONP1
CLPP
YME1L1
CLPX
HSPA9
AFG3L2
GRPEL2
TRAP1
DNAJA3
HSPE1
HSPD1
0.8
0.6
0.4
0.2
0
−0.2
−0.4

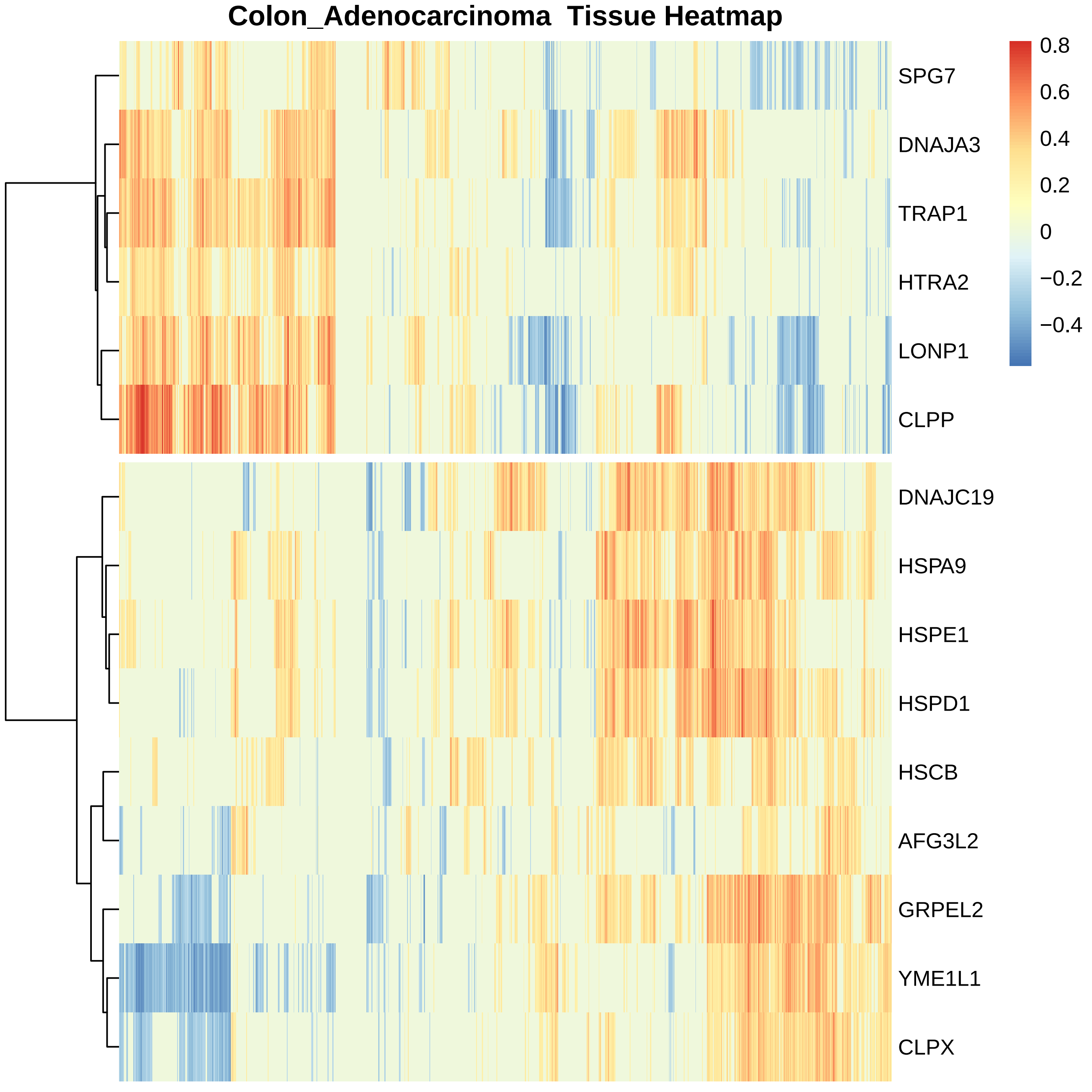
Colon_Adenocarcinoma Tissue Heatmap
SPG7
DNAJA3
TRAP1
HTRA2
LONP1
CLPP
DNAJC19
HSPA9
HSPE1
HSPD1
HSCB
AFG3L2
GRPEL2
YME1L1
CLPX
0.8
0.6
0.4
0.2
0
−0.2
−0.4

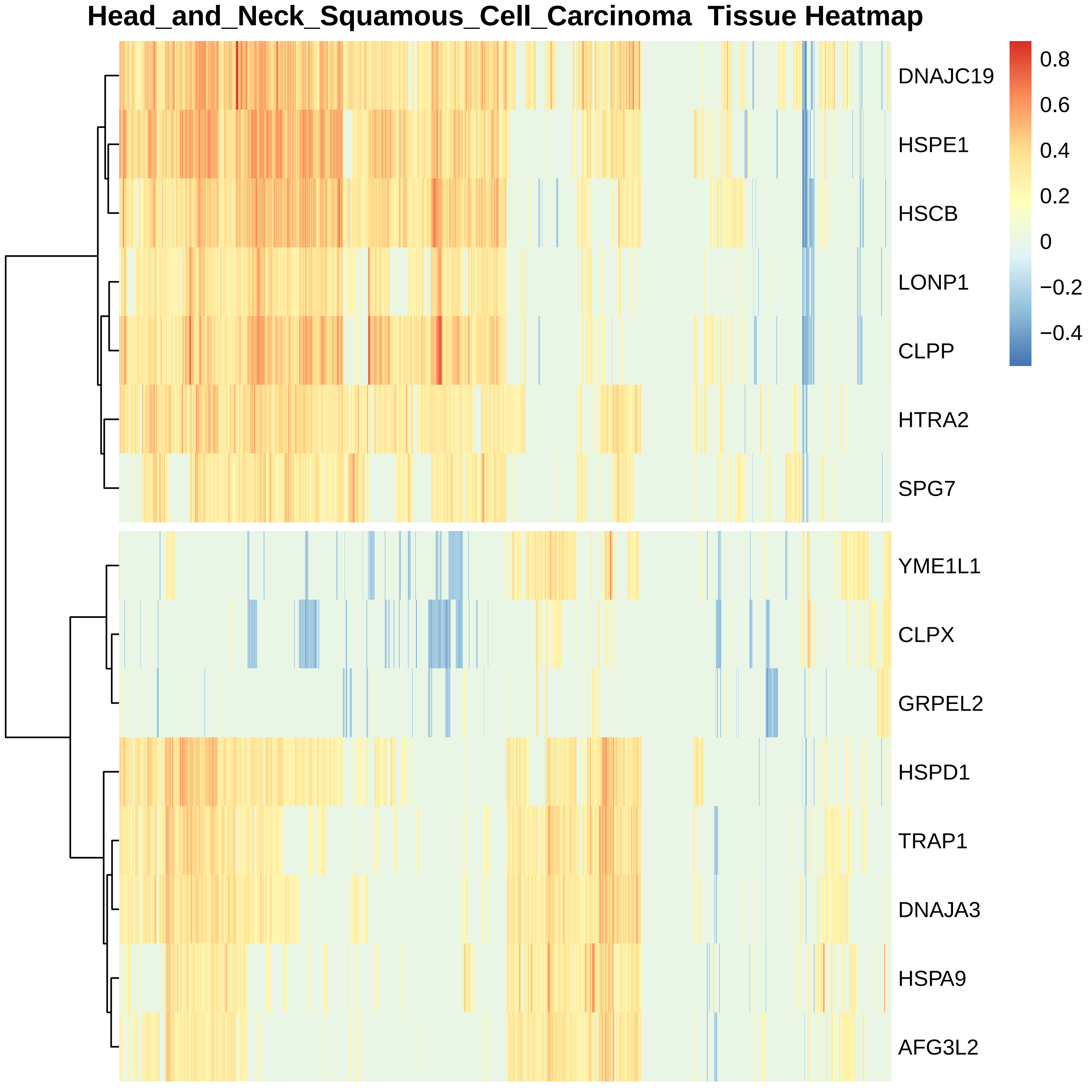
Head_and_Neck_Squamous_Cell_Carcinoma Tissue Heatmap
DNAJC19
HSPE1
HSCB
LONP1
CLPP
HTRA2
SPG7
YME1L1
CLPX
GRPEL2
HSPD1
TRAP1
DNAJA3
HSPA9
AFG3L2
0.8
0.6
0.4
0.2
0
−0.2
−0.4

Kidney_Chromophobe Tissue Heatmap

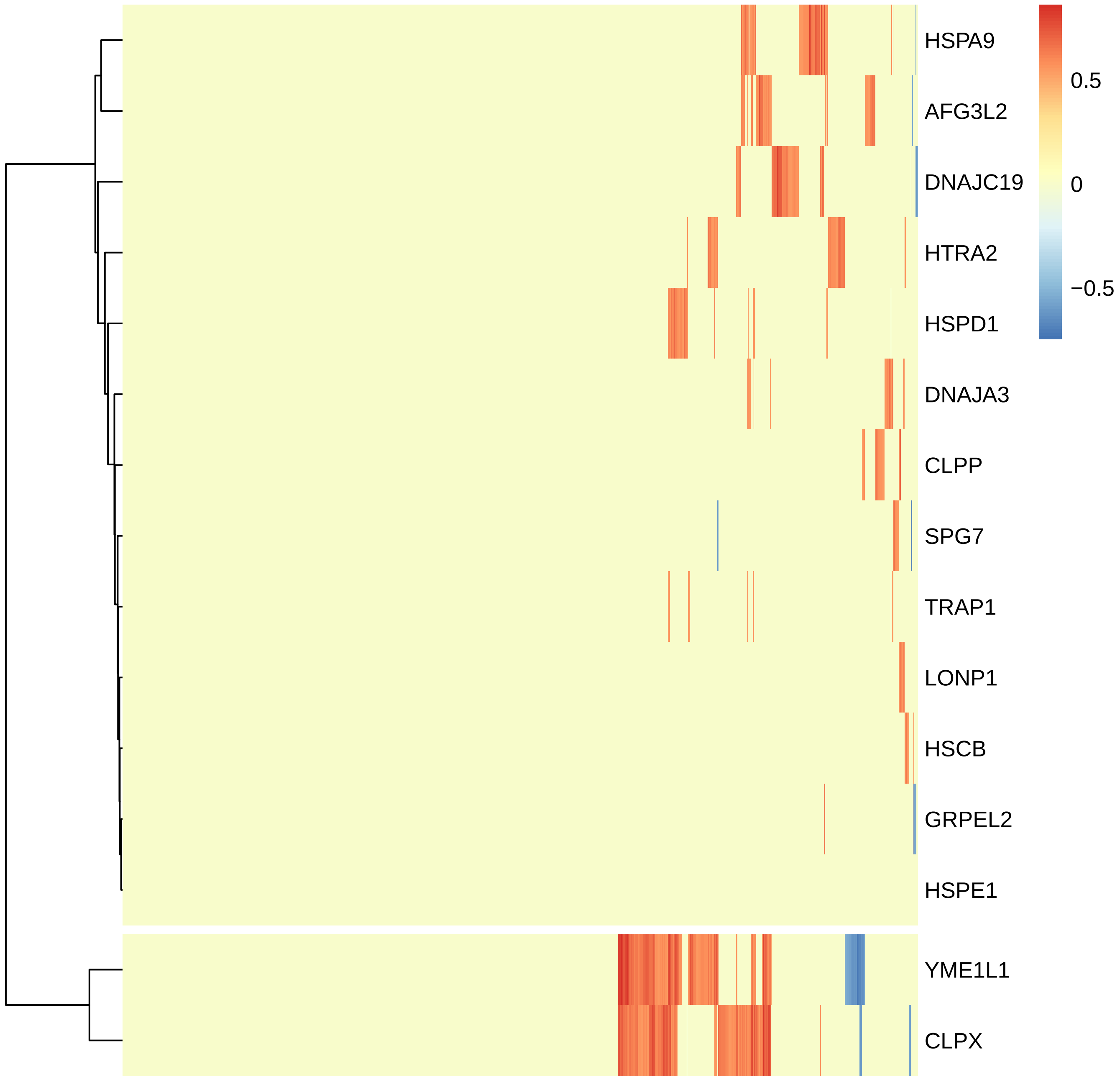

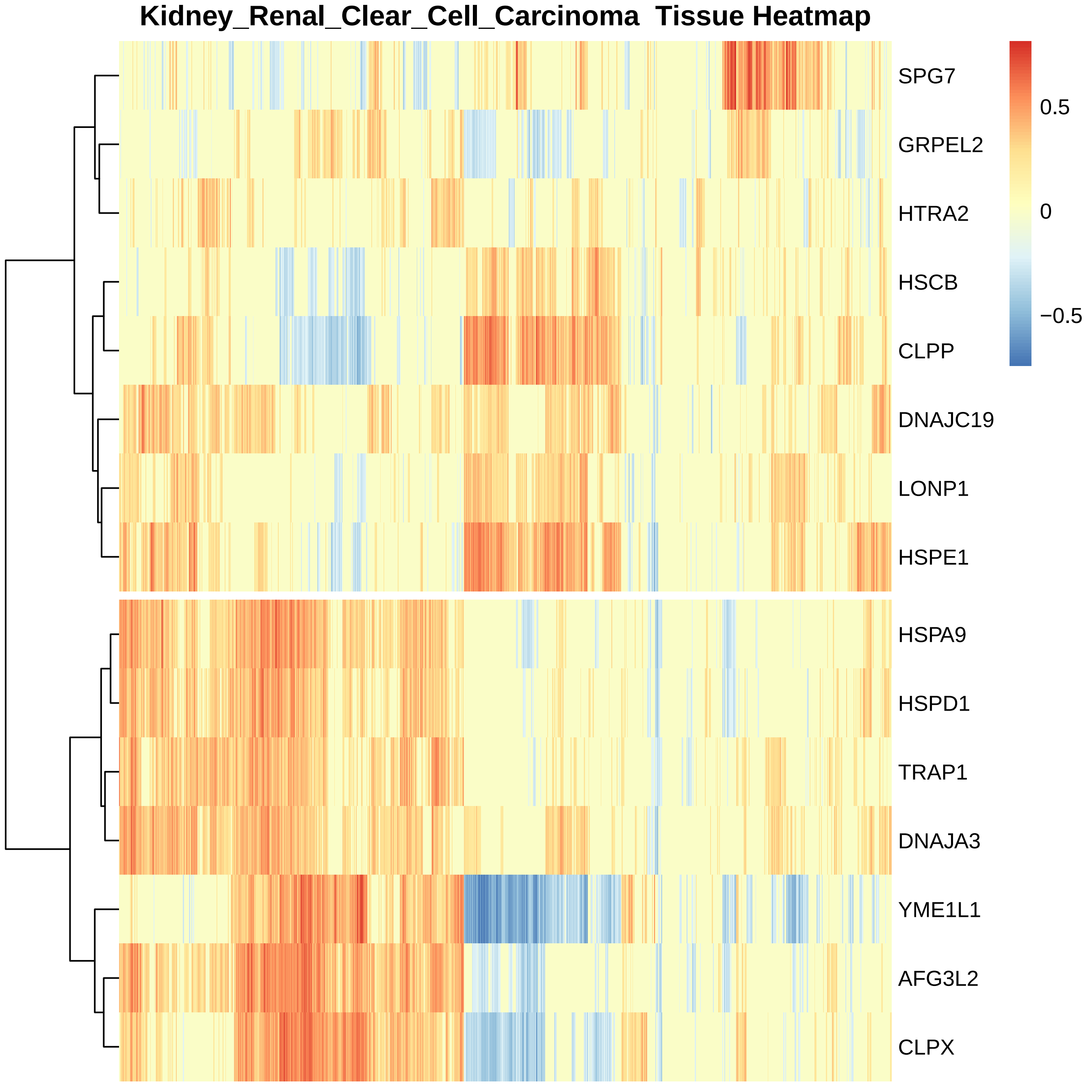

Kidney_Renal_Clear_Cell_Carcinoma Tissue Heatmap
SPG7
GRPEL2
HTRA2
HSCB
CLPP
DNAJC19
LONP1
HSPE1
HSPA9
HSPD1
TRAP1
DNAJA3
YME1L1
AFG3L2
CLPX
0.5
0
−0.5

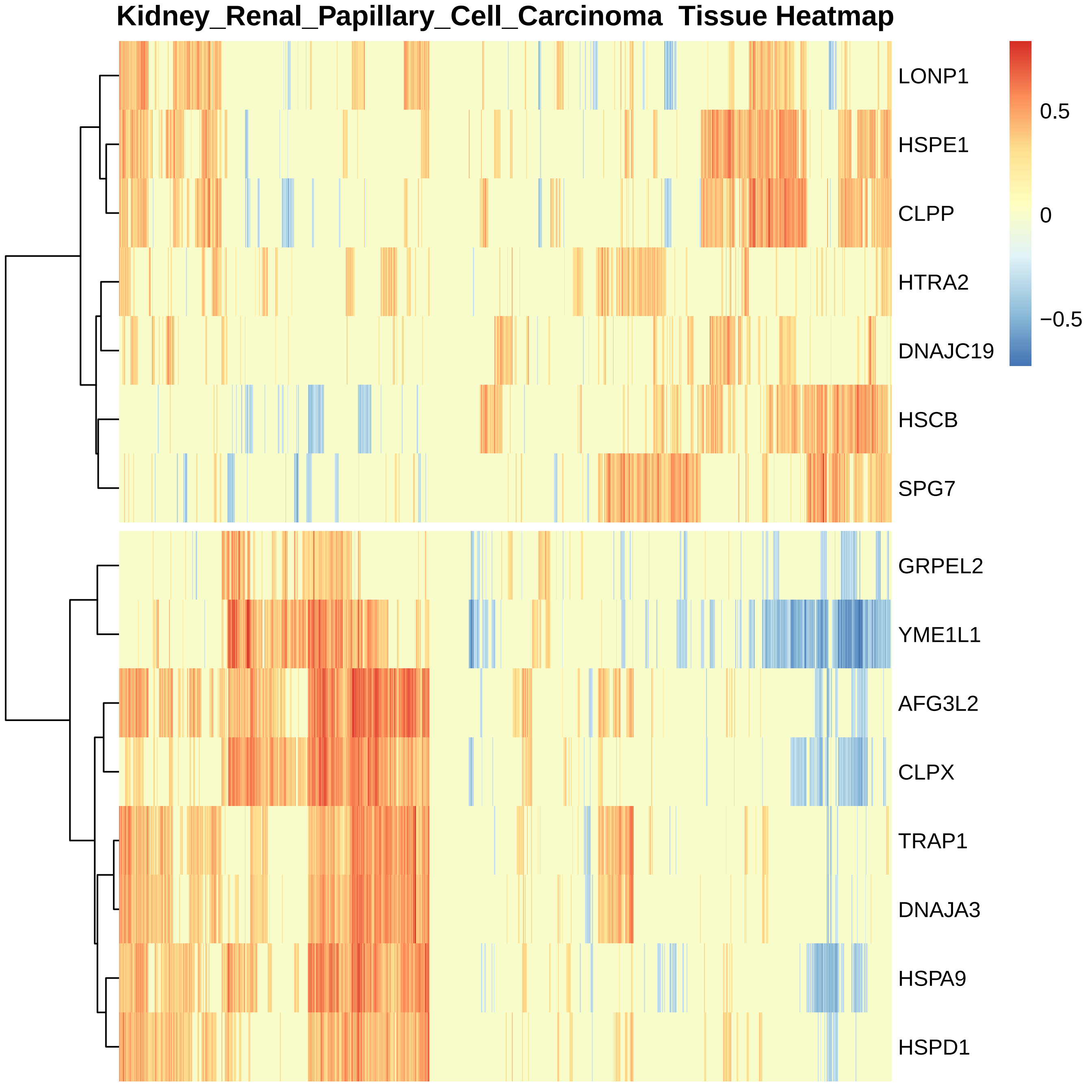

Kidney_Renal_Papillary_Cell_Carcinoma Tissue Heatmap
LONP1
HSPE1
CLPP
HTRA2
DNAJC19
HSCB
SPG7
GRPEL2
YME1L1
AFG3L2
CLPX
TRAP1
DNAJA3
HSPA9
HSPD1
0.5
0
−0.5

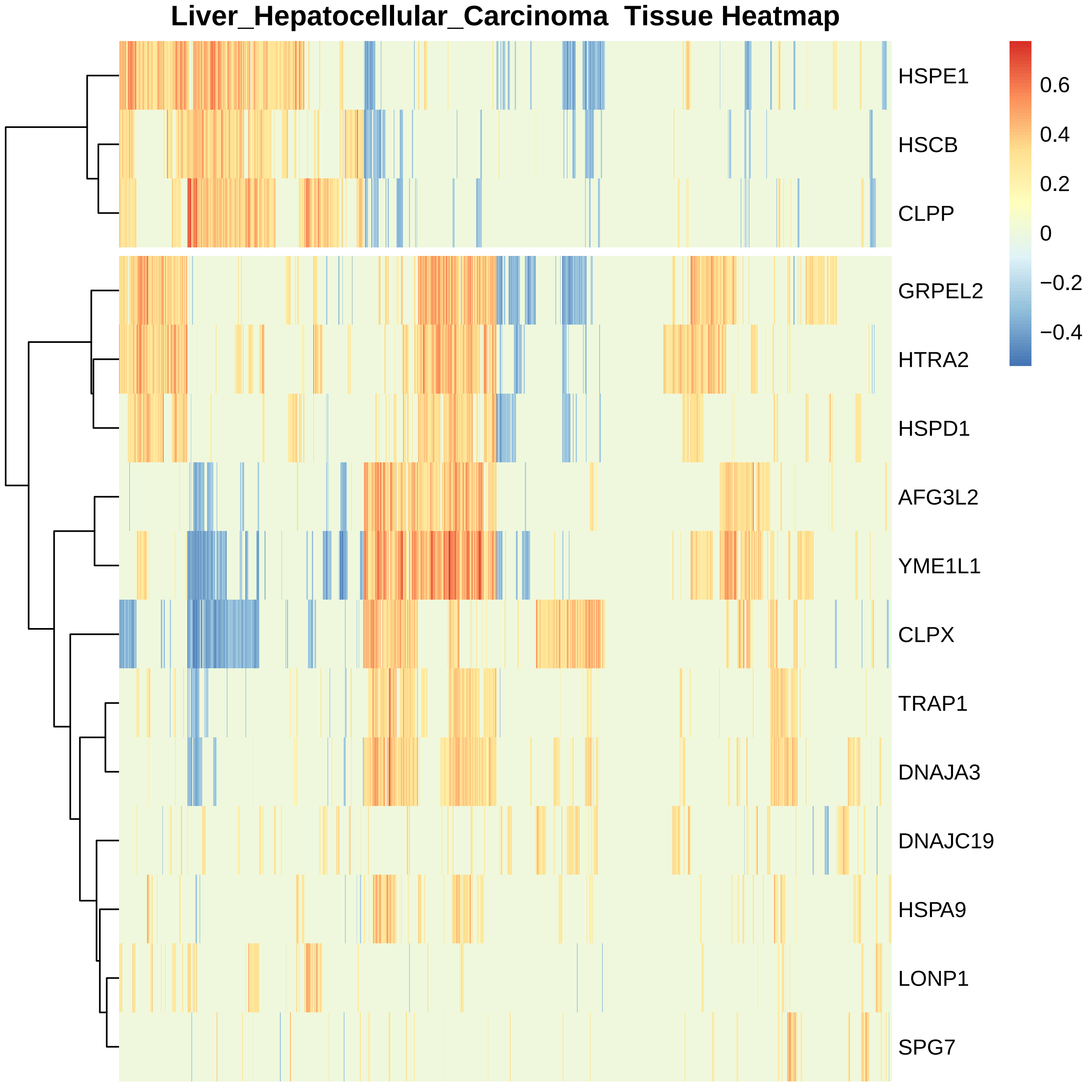
Liver_Hepatocellular_Carcinoma Tissue Heatmap
HSPE1
HSCB
CLPP
GRPEL2
HTRA2
HSPD1
AFG3L2
YME1L1
CLPX
TRAP1
DNAJA3
DNAJC19
HSPA9
LONP1
SPG7
0.6
0.4
0.2
0
−0.2
−0.4

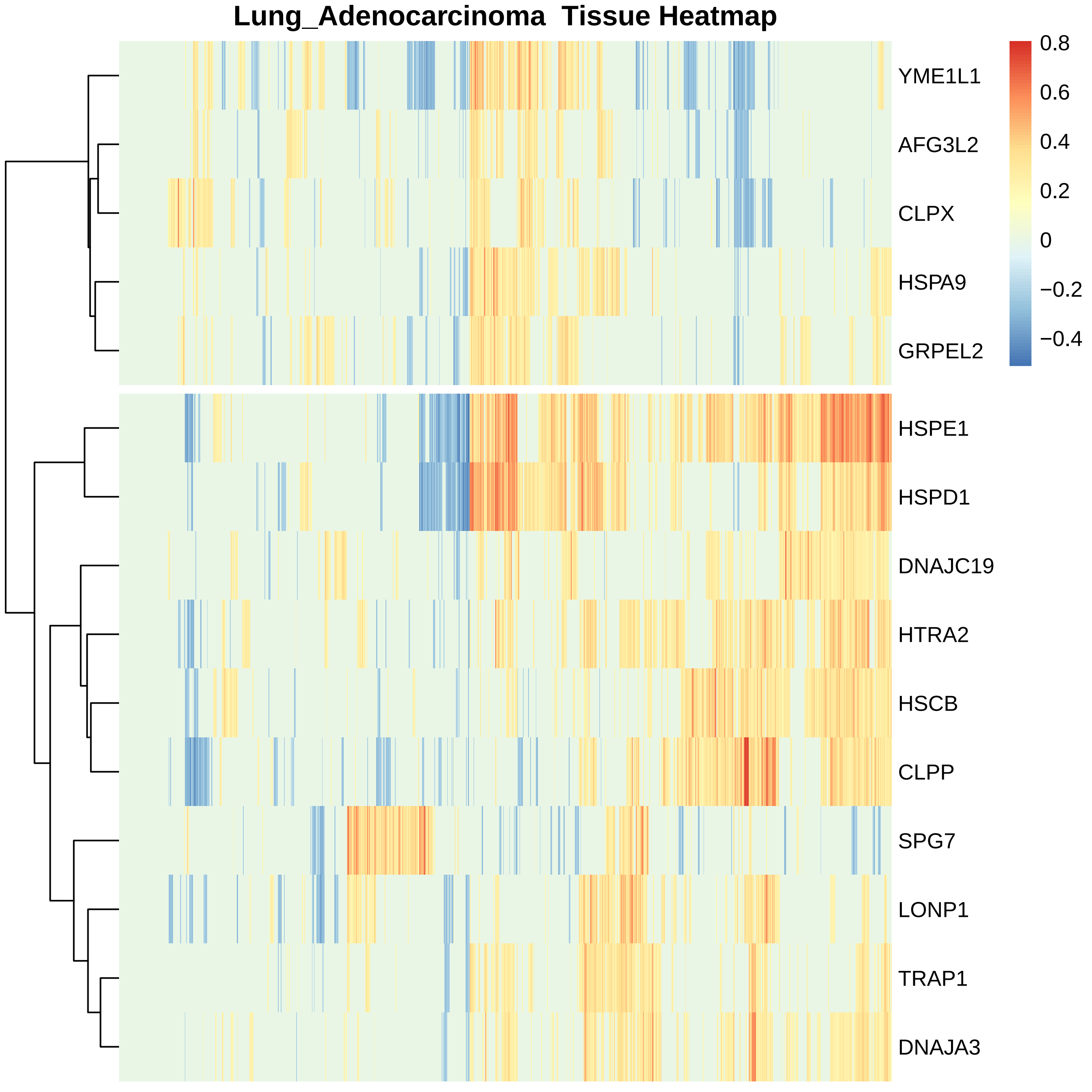
Lung_Adenocarcinoma Tissue Heatmap
YME1L1
AFG3L2
CLPX
HSPA9
GRPEL2
HSPE1
HSPD1
DNAJC19
HTRA2
HSCB
CLPP
SPG7
LONP1
TRAP1
DNAJA3
0.8
0.6
0.4
0.2
0
−0.2
−0.4

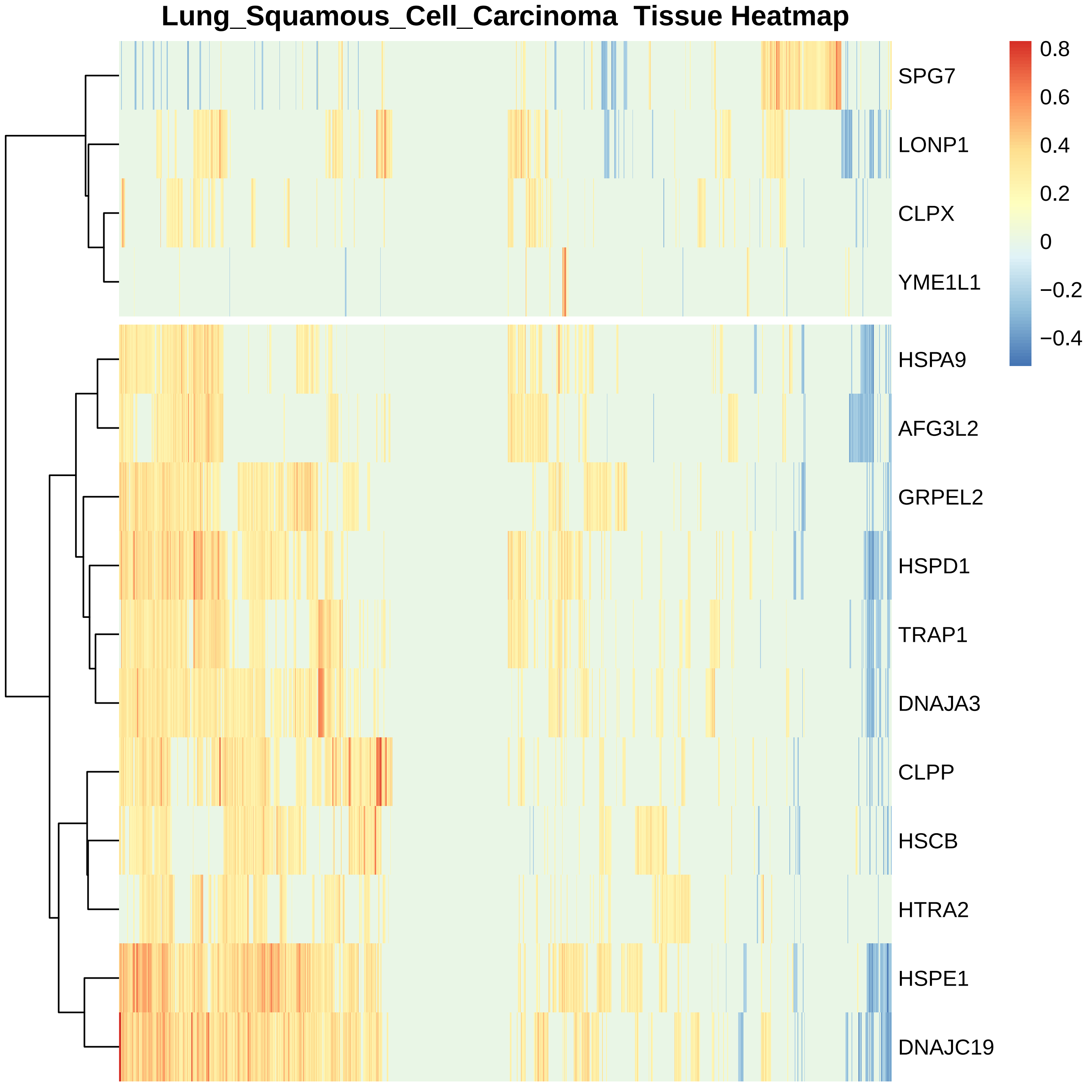
Lung_Squamous_Cell_Carcinoma Tissue Heatmap
SPG7
LONP1
CLPX
YME1L1
HSPA9
AFG3L2
GRPEL2
HSPD1
TRAP1
DNAJA3
CLPP
HSCB
HTRA2
HSPE1
DNAJC19
0.8
0.6
0.4
0.2
0
−0.2
−0.4

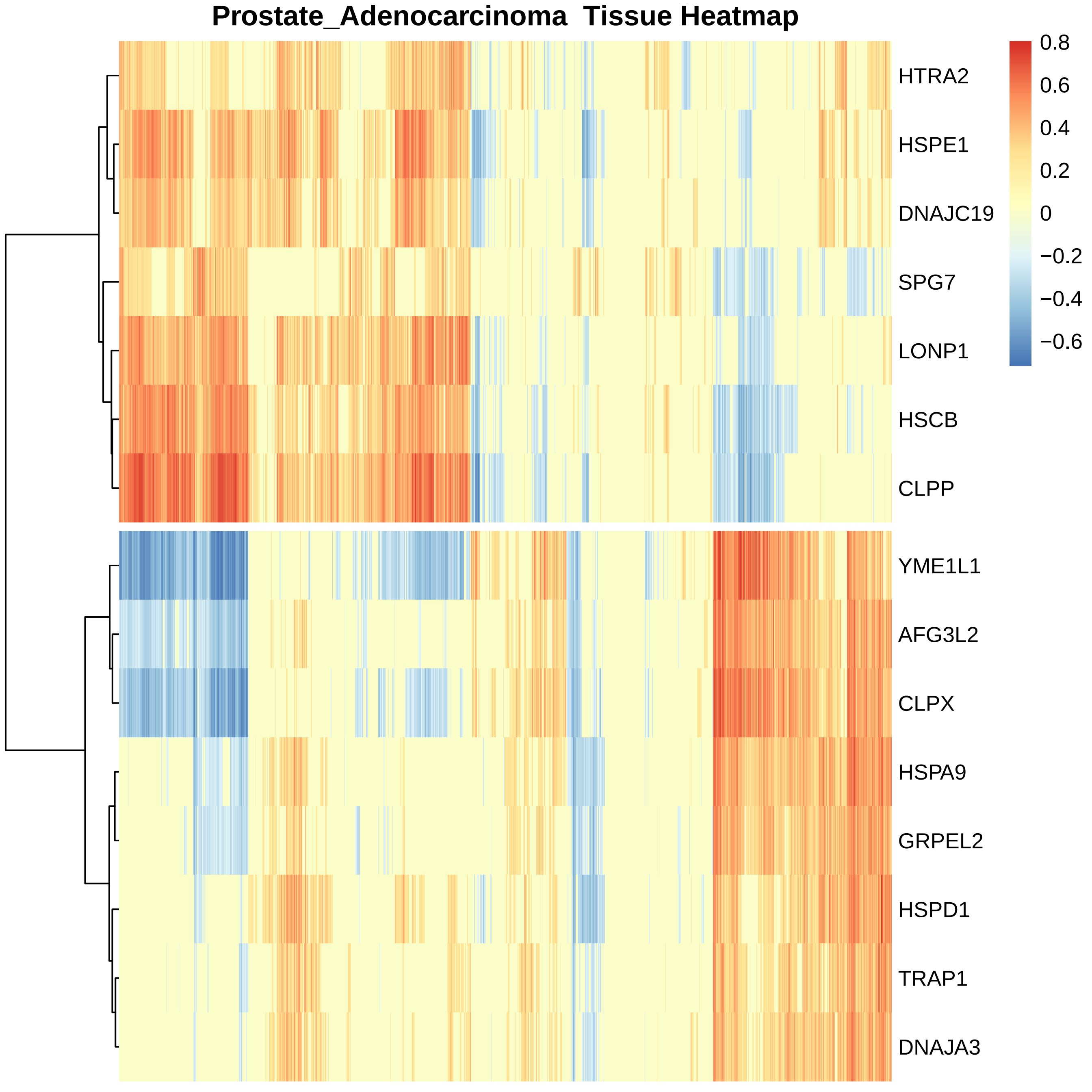

Prostate_Adenocarcinoma Tissue Heatmap
HTRA2
HSPE1
DNAJC19
SPG7
LONP1
HSCB
CLPP
YME1L1
AFG3L2
CLPX
HSPA9
GRPEL2
HSPD1
TRAP1
DNAJA3
0.8
0.6
0.4
0.2
0
−0.2
−0.4
−0.6

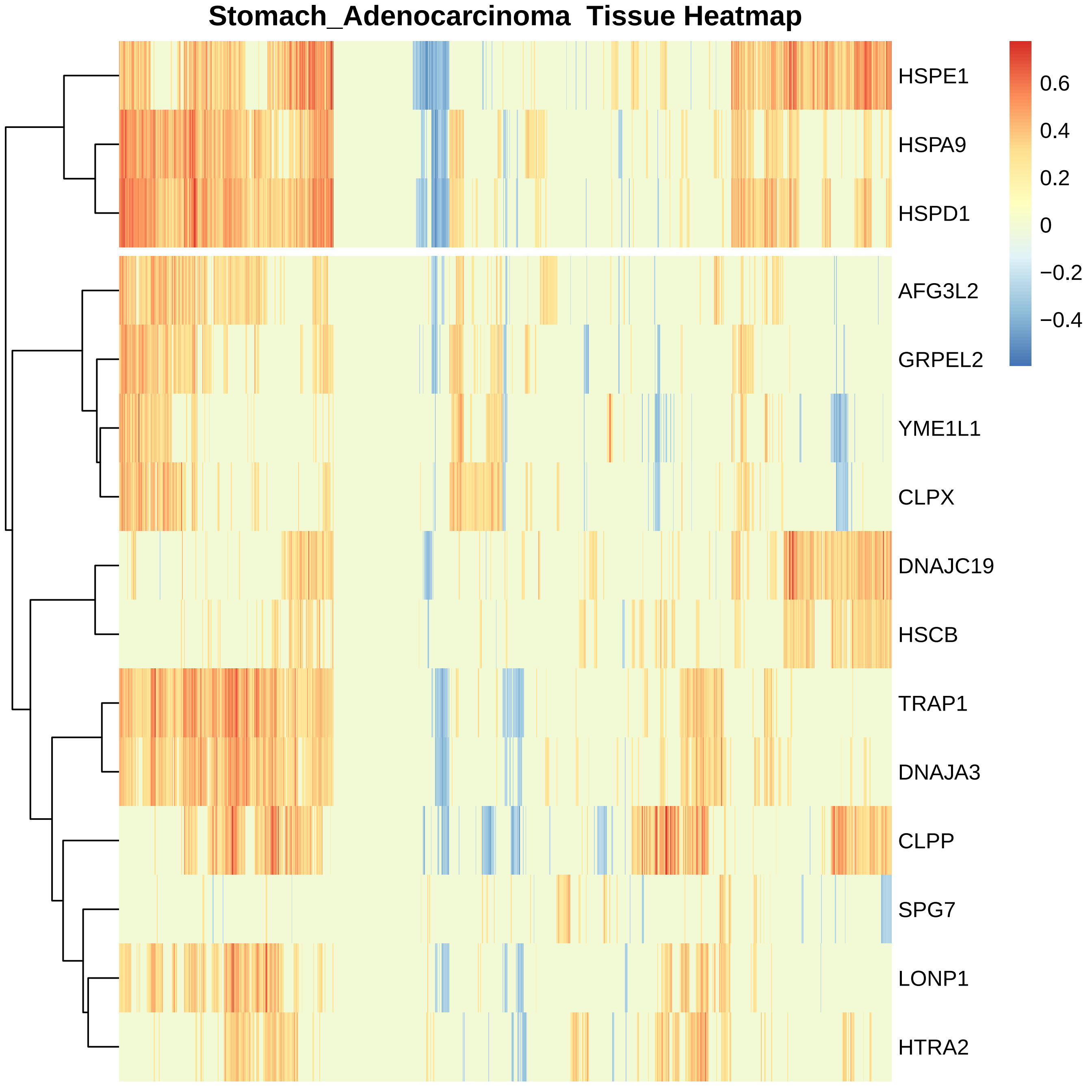
Stomach_Adenocarcinoma Tissue Heatmap
HSPE1
HSPA9
HSPD1
AFG3L2
GRPEL2
YME1L1
CLPX
DNAJC19
HSCB
TRAP1
DNAJA3
CLPP
SPG7
LONP1
HTRA2
0.6
0.4
0.2
0
−0.2
−0.4

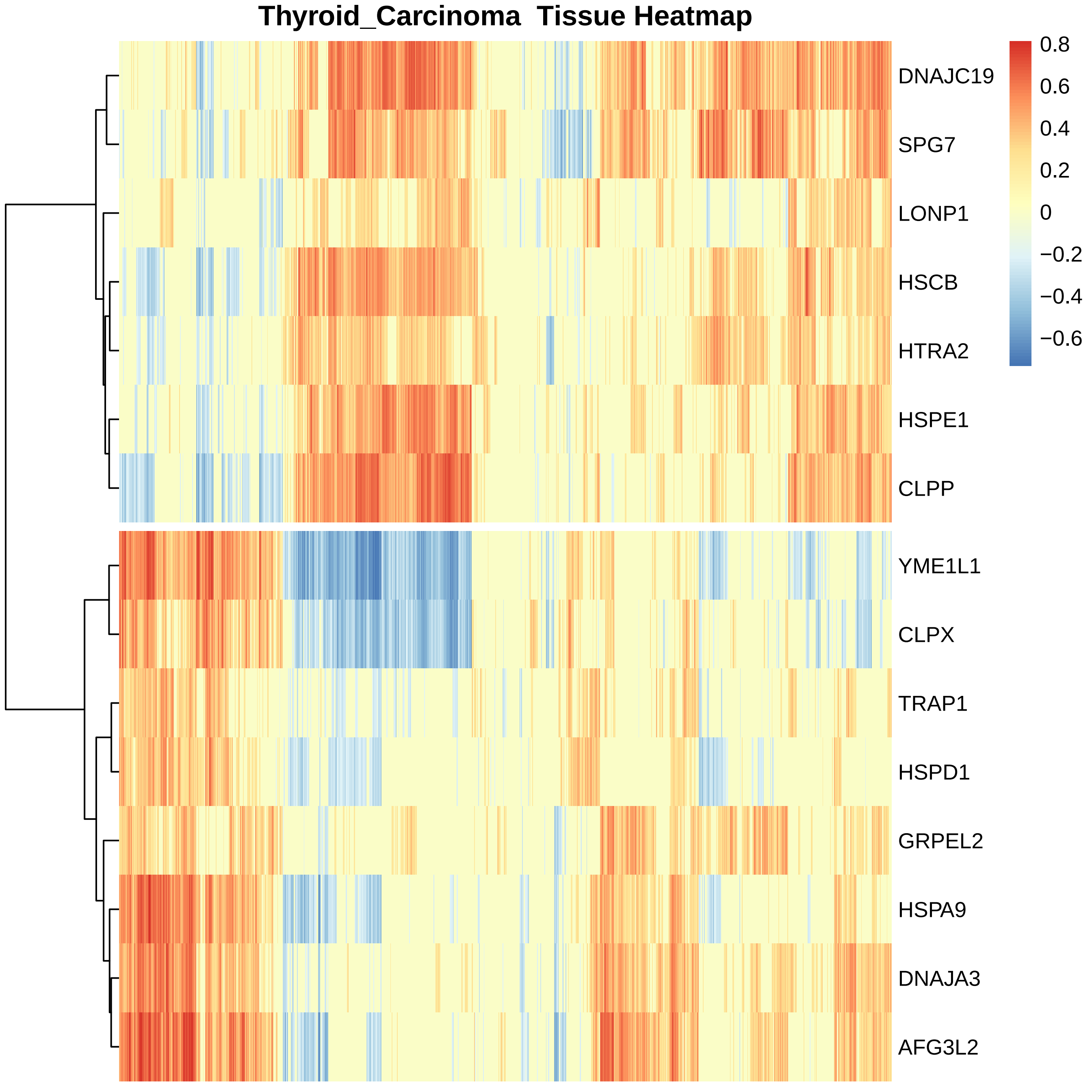
Thyroid_Carcinoma Tissue Heatmap
DNAJC19
SPG7
LONP1
HSCB
HTRA2
HSPE1
CLPP
YME1L1
CLPX
TRAP1
HSPD1
GRPEL2
HSPA9
DNAJA3
AFG3L2
0.8
0.6
0.4
0.2
0
−0.2
−0.4
−0.6

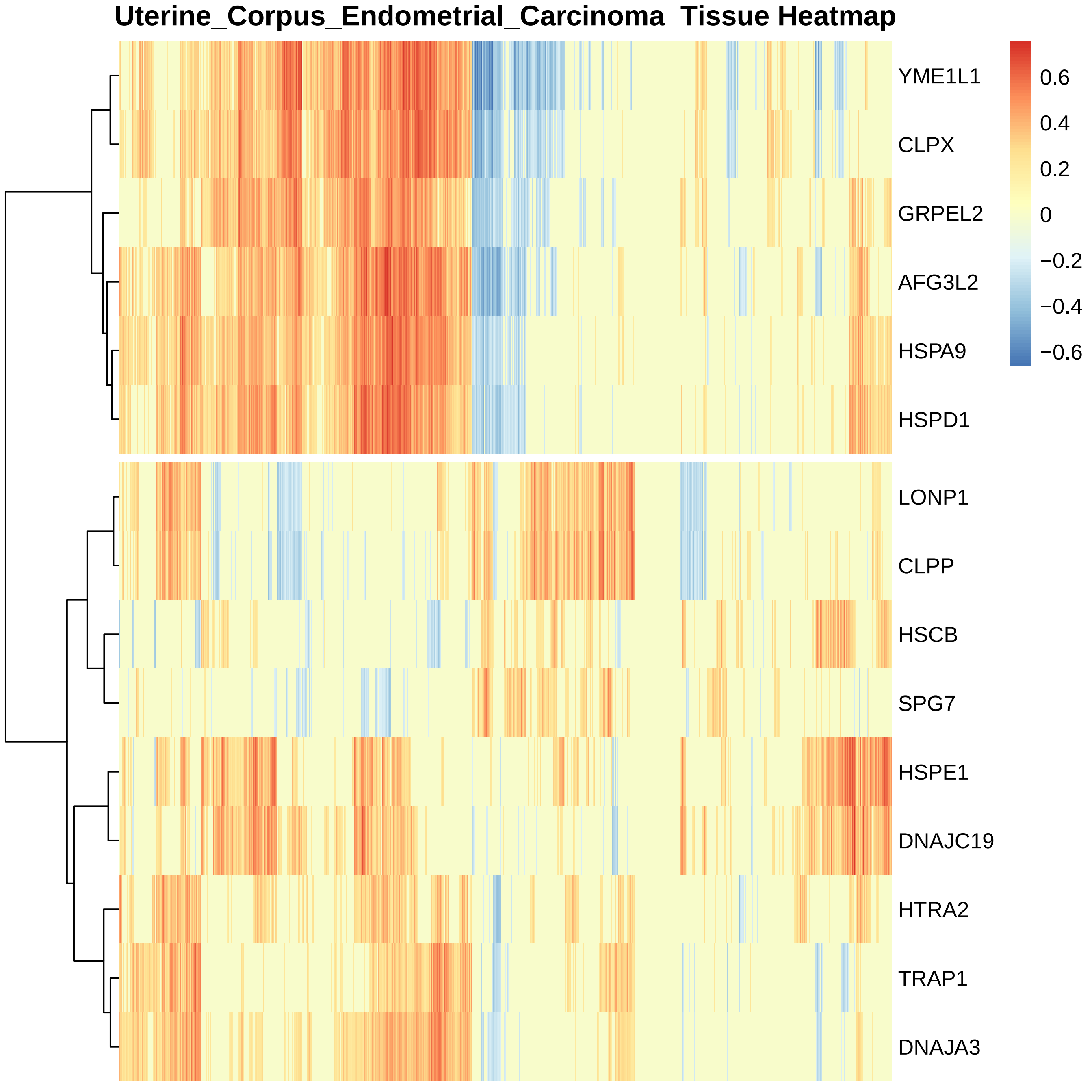
Uterine_Corpus_Endometrial_Carcinoma Tissue Heatmap
YME1L1
CLPX
GRPEL2
AFG3L2
HSPA9
HSPD1
LONP1
CLPP
HSCB
SPG7
HSPE1
DNAJC19
HTRA2
TRAP1
DNAJA3
0.6
0.4
0.2
0
−0.2
−0.4
−0.6
