# Supplementary figures and images for: Pan-Cancer Analysis of Mitochondria Chaperone-Client Co-Expression Reveals Chaperone Functional Partitioning
Source: Cancers (Basel). 2020 Mar 30;12(4):825. doi: 10.3390/cancers12040825 (PMC7226338; doi:10.3390/cancers12040825)

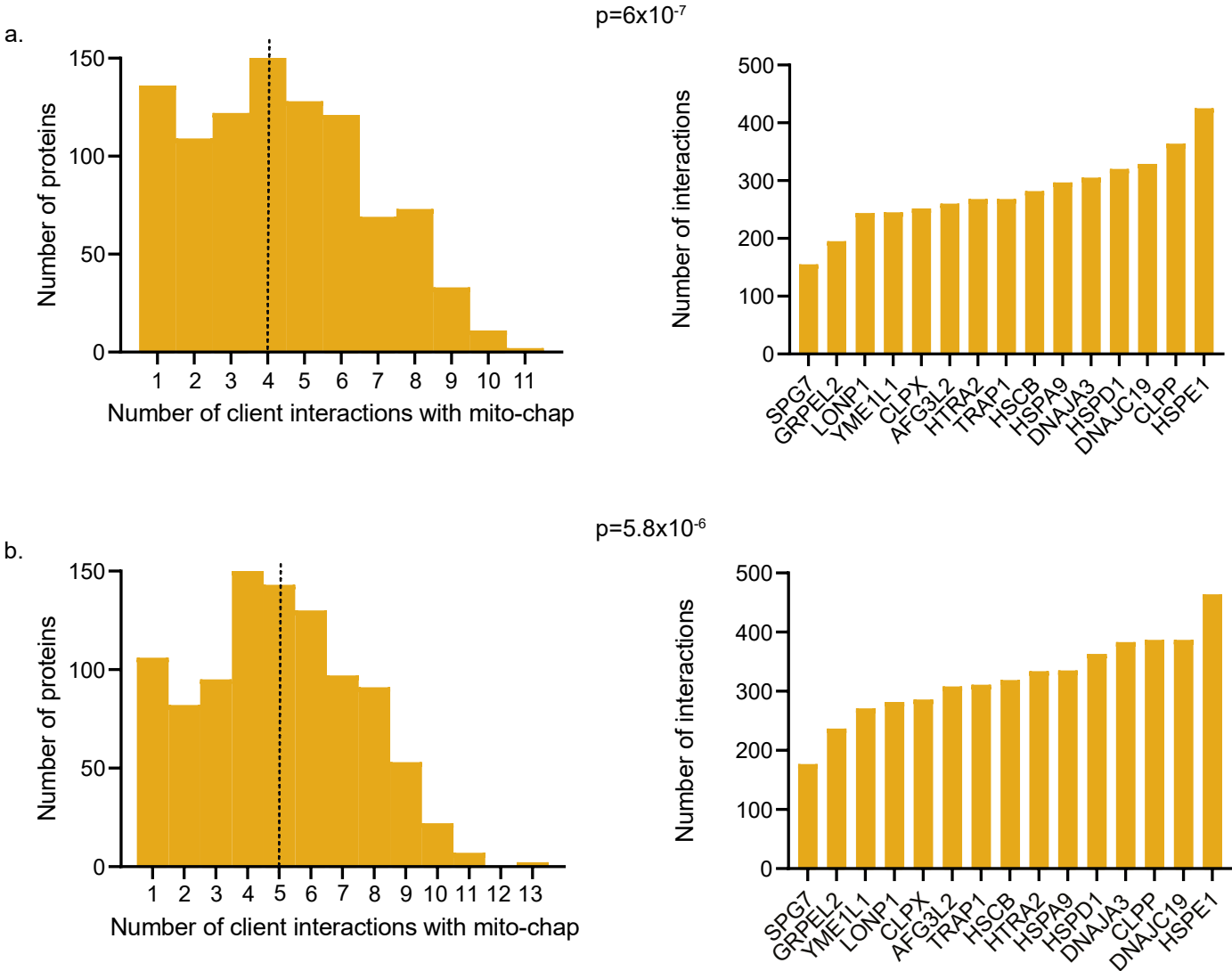

Supplement: Supplementary file 1 [file cancers-12-00825-s001.zip › cancers-735738-supplementary/Galai_et_al_mito-chap - Revised Supplementary Files/Supplementary Figures/SUP_fig_03-p-values.pdf]

Count of R Values in Cancer

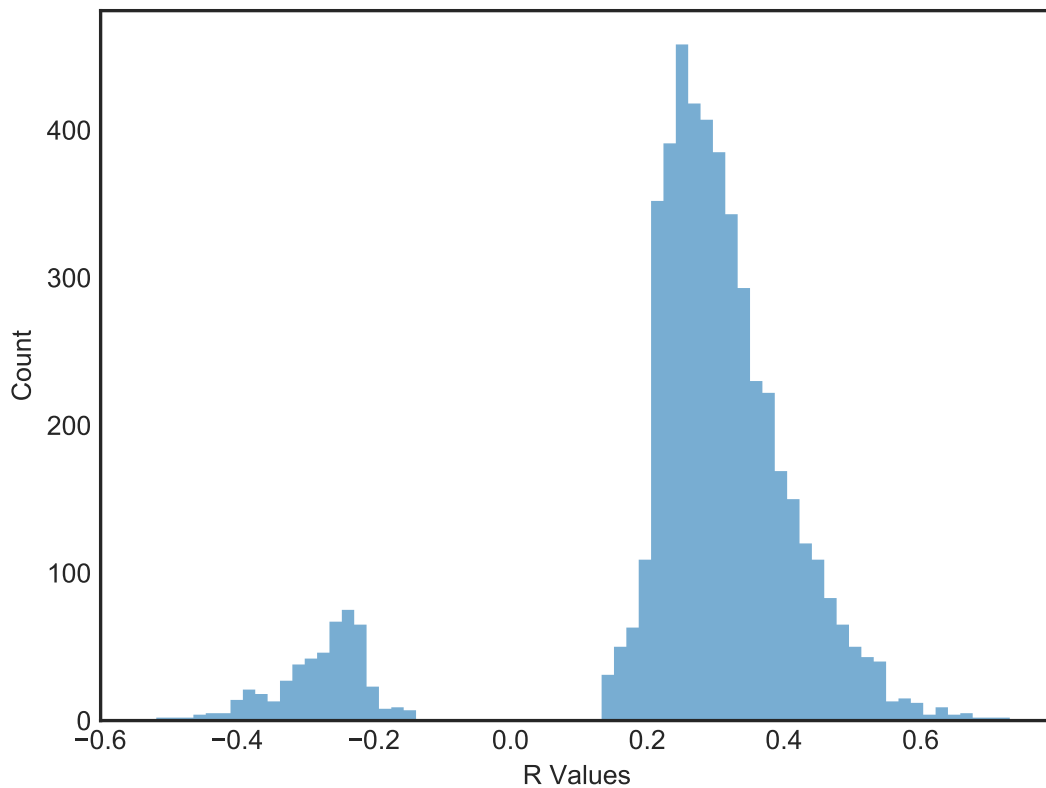

Supplement: Supplementary file 1 [file cancers-12-00825-s001.zip › cancers-735738-supplementary/Galai_et_al_mito-chap - Revised Supplementary Files/Supplementary Figures/SUP_Fig_1-pan_r_histogram.pdf]

# Frequency histogram of simulated L values

observed L value: 7.47

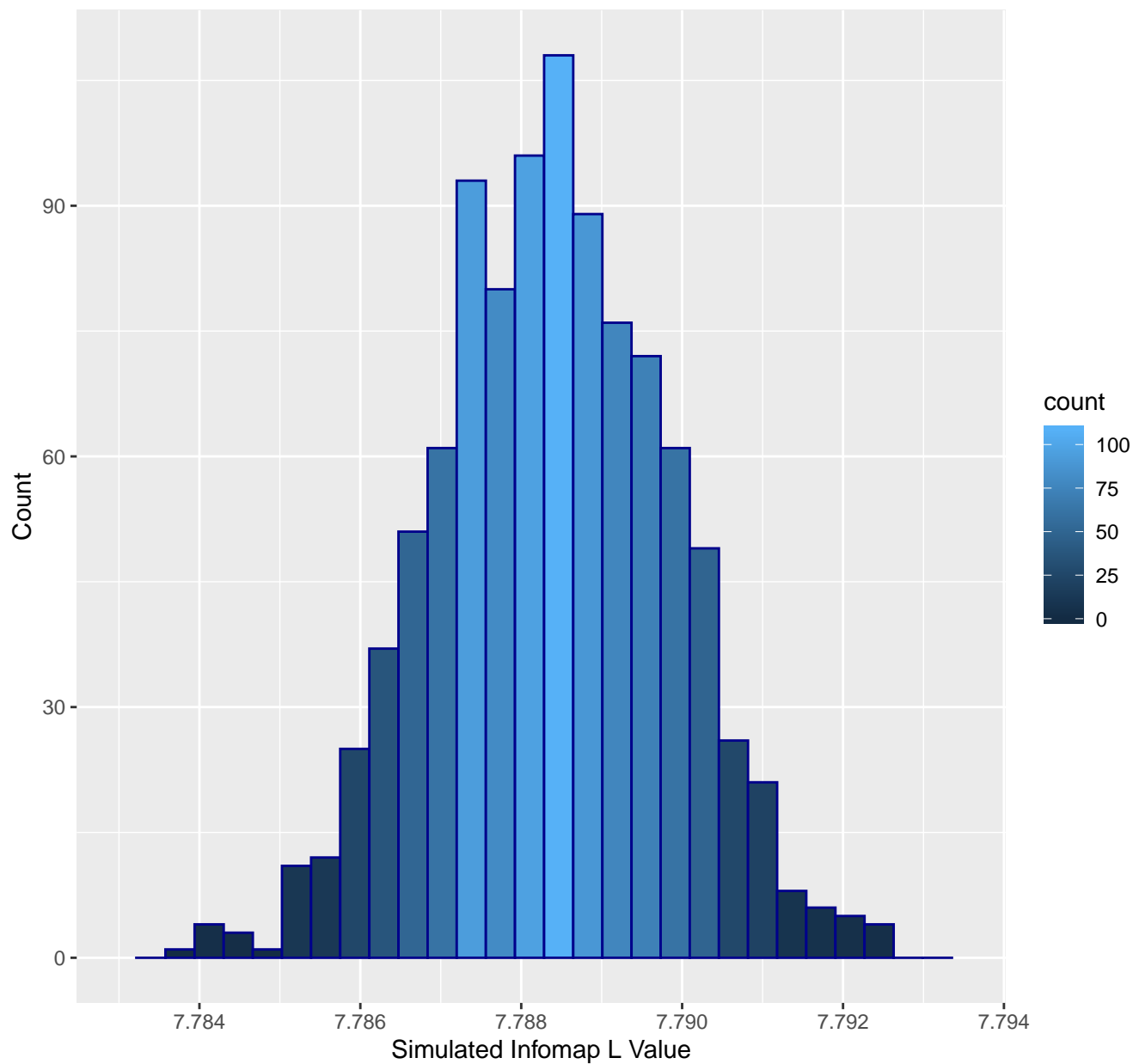

Supplement: Supplementary file 1 [file cancers-12-00825-s001.zip › cancers-735738-supplementary/Galai_et_al_mito-chap - Revised Supplementary Files/Supplementary Figures/SUP_Fig_4_randomization.pdf]
